# [μ-Bis(di-*o*-tolyl­phosphan­yl)methane-1:2κ^2^ *P*:*P*′]nona­carbonyl-1κ^3^ *C*,2κ^3^ *C*,3κ^3^ *C*-[tris­(2-chloro­eth­yl) phosphite-3κ*P*]-*triangulo*-triruthenium(0)

**DOI:** 10.1107/S1600536812023707

**Published:** 2012-05-31

**Authors:** Omar bin Shawkataly, Imthyaz Ahmed Khan, Siti Syaida Sirat, Ching Kheng Quah, Hoong-Kun Fun

**Affiliations:** a Chemical Sciences Programme, School of Distance Education, Universiti Sains Malaysia, 11800 USM, Penang, Malaysia; b X-ray Crystallography Unit, School of Physics, Universiti Sains Malaysia, 11800 USM, Penang, Malaysia

## Abstract

In the title compound, [Ru_3_(C_6_H_12_Cl_3_O_3_P)(C_29_H_30_P_2_)(CO)_9_], the bis­(di-*o*-tolyl­phosphan­yl)methane ligand bridges one Ru—Ru bond and the monodentate phosphite ligand bonds to the third Ru atom. Both ligands are equatorial with respect to the Ru_3_ triangle. Each Ru atom bears one equatorial and two axial terminal carbonyl ligands. The dihedral angles between the two benzene rings in the diphenyl­phosphanyl groups are 79.52 (10) and 69.88 (10)°. In the crystal, mol­ecules are linked *via* C—H⋯O hydrogen bonds into chains along [100].

## Related literature

For general background to *triangulo*-triruthenium compounds with general structure Ru_3_(CO)_12-*n*_ *L*_*n*_ (*L* = group 15 ligand) see: Bruce *et al.* (1985 ▶,1988*a* ▶,*b* ▶); Shawkataly *et al.* (1998 ▶, 2004 ▶, 2010 ▶, 2011 ▶). For the preparation of the di-*o*-tolyl­phosphan­yl ligand, see: Filby *et al.* (2006 ▶). For the stability of the temperature controller used in the data collection, see: Cosier & Glazer (1986 ▶).
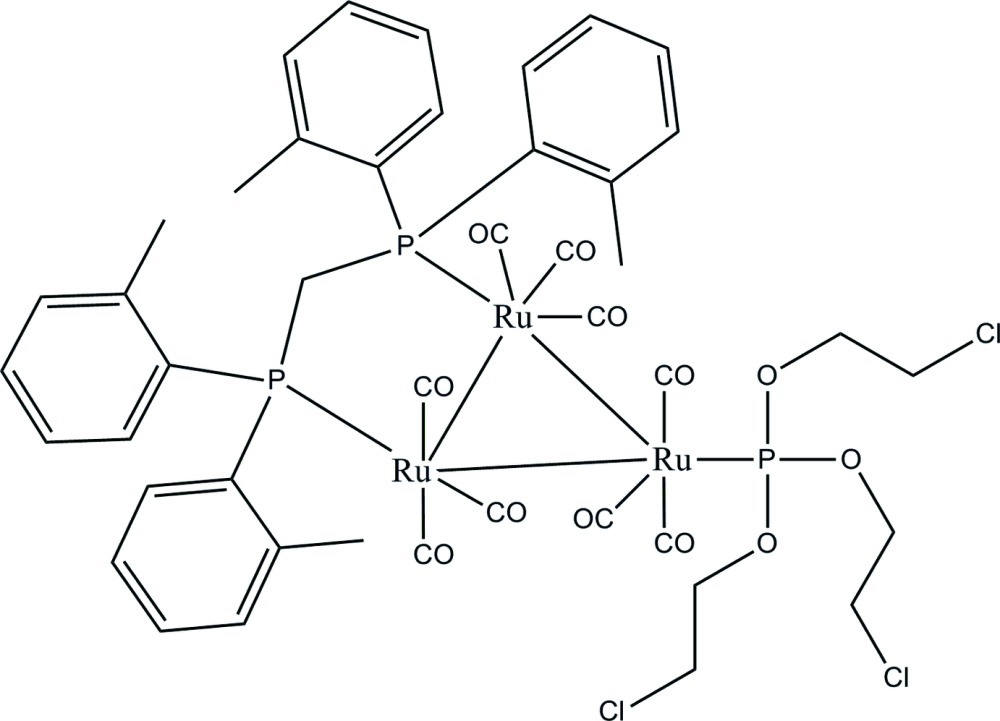


## Experimental

### 

#### Crystal data

[Ru_3_(C_6_H_12_Cl_3_O_3_P)(C_29_H_30_P_2_)(CO)_9_]
*M* *_r_* = 1265.25
Monoclinic, 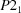

*a* = 10.1705 (6) Å
*b* = 20.7490 (12) Å
*c* = 12.3584 (7) Å
β = 109.241 (1)°
*V* = 2462.3 (2) Å^3^
*Z* = 2
Mo *K*α radiation
μ = 1.23 mm^−1^
*T* = 100 K
0.63 × 0.30 × 0.09 mm

#### Data collection

Bruker SMART APEXII DUO CCD area-detector diffractometer
Absorption correction: multi-scan (*SADABS*; Bruker, 2009 ▶) *T*_min_ = 0.513, *T*_max_ = 0.899
23611 measured reflections
11176 independent reflections
11085 reflections with *I* > 2σ(*I*)
*R*_int_ = 0.016

#### Refinement

*R*[*F* ^2^ > 2σ(*F* ^2^)] = 0.016
*wR*(*F* ^2^) = 0.042
*S* = 1.05
11176 reflections
590 parameters
1 restraint
H-atom parameters constrained
Δρ_max_ = 0.78 e Å^−3^
Δρ_min_ = −0.50 e Å^−3^
Absolute structure: Flack (1983 ▶), 5368 Friedel pairs
Flack parameter: 0.016 (10)

### 

Data collection: *APEX2* (Bruker, 2009 ▶); cell refinement: *SAINT* (Bruker, 2009 ▶); data reduction: *SAINT*; program(s) used to solve structure: *SHELXTL* (Sheldrick, 2008 ▶); program(s) used to refine structure: *SHELXTL*; molecular graphics: *SHELXTL*; software used to prepare material for publication: *SHELXTL* and *PLATON* (Spek, 2009 ▶).

## Supplementary Material

Crystal structure: contains datablock(s) global, I. DOI: 10.1107/S1600536812023707/rz2762sup1.cif

Structure factors: contains datablock(s) I. DOI: 10.1107/S1600536812023707/rz2762Isup2.hkl

Additional supplementary materials:  crystallographic information; 3D view; checkCIF report

## Figures and Tables

**Table 1:** Hydrogen-bond geometry (Å, °)

*D*—H⋯*A*	*D*—H	H⋯*A*	*D*⋯*A*	*D*—H⋯*A*
C11—H11*A*⋯O1^i^	0.93	2.57	3.204 (3)	126

Symmetry code: (i) 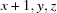.

**Fig. 1.**
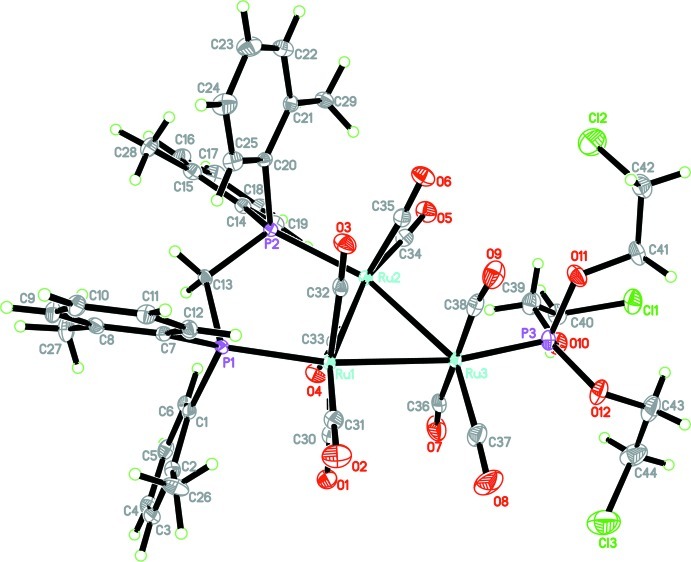
The molecular structure of the title compound showing 50% probability displacement ellipsoids for non-H atoms.

**Fig. 2.**
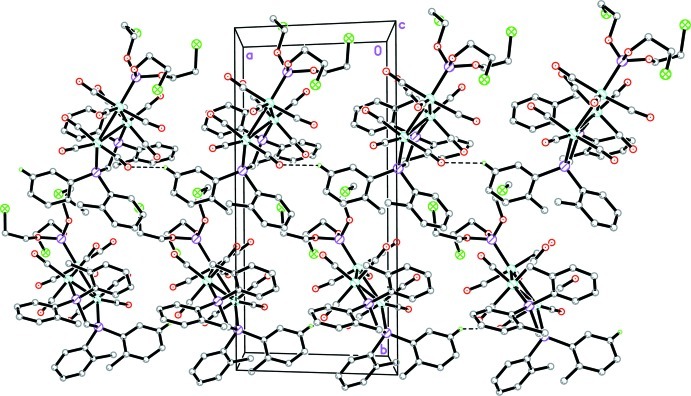
The crystal structure of the title compound, viewed down the c axis. H atoms not involved in hydrogen bonds (dashed lines) have been omitted for clarity.

## supplementary crystallographic information

### Comment

A large number of substituted derivatives of the type Ru_3_(CO)_12-_*_n_**L**_n_* (*L* = group 15 ligand) have been reported (Bruce *et al.*,1985, 1988*a*,*b*). As part of our study on the substitution of transition metal-carbonyl clusters with mixed-ligand complexes, we have published several structures of *triangulo*-triruthenium-carbonyl clusters containing mixed P/As and P/Sb ligands (Shawkataly *et al.*, 1998, 2004, 2010, 2011). Herein we report the synthesis and structure of the title compound.

In the title triangulo-triruthenium compound, the bis(di-*o*-tolylphosphanyl)methane ligand bridges the Ru1–Ru2 bond and the monodentate phosphite ligand bonds to the Ru3 atom. Both phosphorous ligands are equatorial with respect to the Ru_3_ triangle. Moreover, each Ru atom carries one equatorial and two axial terminal carbonyl ligands (Fig. 1). The dihedral angles between the two benzene rings (C1–C6/C7–C12 and C14–C19/C20–C25) are 79.52 (10) and 69.88 (10)° for the two diphenylphosphanyl groups respectively.

In the crystal structure, Fig. 2, molecules are linked *via* intermolecular C11–H11A···O1 hydrogen bonds (Table 1) into one-dimensional chains along [100].

### Experimental

All manipulations were performed under a dry oxygen-free nitrogen atmosphere using standard Schlenk techniques. All solvents were dried over sodium and distilled from sodium benzophenone ketyl under dry oxygen free nitrogen. Tris(2-chloroethyl)phosphite (Aldrich) was used as received and bis(di-*o*-tolylphosphanyl)methane (Filby *et al.*, 2006) was prepared by reported procedure. Ru_3_(CO)_10_(µ-(2-CH_3_C_6_H_4_)_2_PCH_2_P(2-CH_3_C_6_H_4_)_2_) was prepared by reacting Ru_3_(CO)_12_ with bis(di-*o*-tolylphosphanyl)methane in presence of sodium benzophenone ketyl radical in THF (Shawkataly *et al.*,2011). The title compound was obtained by refluxing equimolar quantities of Ru_3_(CO)_10_(µ-(2-(CH_3_C_6_H_4_)_2_PCH_2_P(2-CH_3_C_6_H_4_)_2_) and tris(2-chloroethyl)phosphite in hexane under nitrogen atmosphere. Crystals suitable for X-ray diffraction were grown by slow solvent/solvent diffusion of CH_3_OH into CH_2_Cl_2_.

### Refinement

All H atoms were positioned geometrically and refined using a riding model with C–H = 0.93 or 0.97 Å and *U*_iso_(H) = 1.2 or 1.5 *U*_eq_(C). A rotating group model was applied to the methyl groups.

### Crystal data

**Table:**

[Ru_3_(C_6_H_12_Cl_3_O_3_P)(C_29_H_30_P_2_)(CO)_9_]	*F*(000) = 1260
*M**_r_* = 1265.25	*D*_x_ = 1.707 Mg m^−^^3^
Monoclinic, *P*2_1_	Mo *K*α radiation, λ = 0.71073 Å
Hall symbol: P 2yb	Cell parameters from 9989 reflections
*a* = 10.1705 (6) Å	θ = 2.9–30.1°
*b* = 20.7490 (12) Å	µ = 1.23 mm^−^^1^
*c* = 12.3584 (7) Å	*T* = 100 K
β = 109.241 (1)°	Plate, brown
*V* = 2462.3 (2) Å^3^	0.63 × 0.30 × 0.09 mm
*Z* = 2	

### Data collection

**Table:**

Bruker SMART APEXII DUO CCD area-detector diffractometer	11176 independent reflections
Radiation source: fine-focus sealed tube	11085 reflections with *I* > 2σ(*I*)
Graphite monochromator	*R*_int_ = 0.016
φ and ω scans	θ_max_ = 27.5°, θ_min_ = 1.8°
Absorption correction: multi-scan (*SADABS*; Bruker, 2009)	*h* = −13→13
*T*_min_ = 0.513, *T*_max_ = 0.899	*k* = −26→26
23611 measured reflections	*l* = −15→16

### Refinement

**Table:**

Refinement on *F*^2^	Secondary atom site location: difference Fourier map
Least-squares matrix: full	Hydrogen site location: inferred from neighbouring sites
*R*[*F*^2^ > 2σ(*F*^2^)] = 0.016	H-atom parameters constrained
*wR*(*F*^2^) = 0.042	*w* = 1/[σ^2^(*F*_o_^2^) + (0.0205*P*)^2^ + 0.6778*P*] where *P* = (*F*_o_^2^ + 2*F*_c_^2^)/3
*S* = 1.05	(Δ/σ)_max_ = 0.001
11176 reflections	Δρ_max_ = 0.78 e Å^−^^3^
590 parameters	Δρ_min_ = −0.50 e Å^−^^3^
1 restraint	Absolute structure: Flack (1983), 5368 Friedel pairs
Primary atom site location: structure-invariant direct methods	Flack parameter: 0.016 (10)

### Special details

**Table:**

Experimental. The crystal was placed in the cold stream of an Oxford Cryosystems Cobra open-flow nitrogen cryostat (Cosier & Glazer, 1986) operating at 100 K.
Geometry. All esds (except the esd in the dihedral angle between two l.s. planes) are estimated using the full covariance matrix. The cell esds are taken into account individually in the estimation of esds in distances, angles and torsion angles; correlations between esds in cell parameters are only used when they are defined by crystal symmetry. An approximate (isotropic) treatment of cell esds is used for estimating esds involving l.s. planes.
Refinement. Refinement of F^2^ against ALL reflections. The weighted R-factor wR and goodness of fit S are based on F^2^, conventional R-factors R are based on F, with F set to zero for negative F^2^. The threshold expression of F^2^ > 2sigma(F^2^) is used only for calculating R-factors(gt) etc. and is not relevant to the choice of reflections for refinement. R-factors based on F^2^ are statistically about twice as large as those based on F, and R- factors based on ALL data will be even larger.

### Fractional atomic coordinates and isotropic or equivalent isotropic displacement parameters (Å^2^)

**Table:**

	*x*	*y*	*z*	*U*_iso_*/*U*_eq_	
Ru1	0.944298 (14)	0.325120 (7)	0.809961 (11)	0.00983 (3)	
Ru2	0.765192 (14)	0.257429 (7)	0.616918 (12)	0.01032 (3)	
Ru3	0.788240 (15)	0.218119 (7)	0.844371 (12)	0.01256 (3)	
Cl1	0.27592 (6)	0.02481 (3)	0.67579 (5)	0.02873 (12)	
Cl2	0.70147 (10)	−0.02232 (4)	0.58668 (6)	0.05110 (19)	
Cl3	0.49238 (9)	0.17761 (4)	1.09900 (7)	0.0544 (2)	
P1	0.97101 (5)	0.41265 (2)	0.69855 (4)	0.01024 (9)	
P2	0.84641 (5)	0.31652 (2)	0.48990 (4)	0.01023 (9)	
P3	0.66767 (5)	0.12666 (3)	0.84199 (4)	0.01523 (10)	
O1	0.72264 (15)	0.40026 (8)	0.87717 (13)	0.0205 (3)	
O2	1.14022 (17)	0.35167 (9)	1.05085 (13)	0.0280 (4)	
O3	1.17618 (15)	0.24273 (7)	0.77020 (13)	0.0210 (3)	
O4	0.55800 (15)	0.36598 (8)	0.61476 (13)	0.0214 (3)	
O5	0.55140 (16)	0.17020 (8)	0.45254 (14)	0.0241 (3)	
O6	0.96944 (16)	0.14721 (8)	0.62476 (13)	0.0222 (3)	
O7	0.50273 (18)	0.28256 (8)	0.79736 (17)	0.0318 (4)	
O8	0.8773 (2)	0.24850 (11)	1.09856 (14)	0.0409 (5)	
O9	1.05262 (17)	0.13615 (9)	0.88318 (15)	0.0279 (4)	
O10	0.50288 (16)	0.12107 (8)	0.77775 (13)	0.0226 (3)	
O11	0.73473 (16)	0.06597 (7)	0.80111 (15)	0.0242 (3)	
O12	0.66222 (17)	0.10944 (8)	0.96670 (14)	0.0262 (3)	
C1	0.86732 (19)	0.48302 (9)	0.71299 (16)	0.0127 (4)	
C2	0.9109 (2)	0.52284 (10)	0.81082 (17)	0.0162 (4)	
C3	0.8240 (2)	0.57358 (10)	0.81935 (18)	0.0177 (4)	
H3A	0.8521	0.5998	0.8840	0.021*	
C4	0.6980 (2)	0.58619 (10)	0.73529 (19)	0.0194 (4)	
H4A	0.6438	0.6209	0.7425	0.023*	
C5	0.6533 (2)	0.54589 (10)	0.63914 (18)	0.0175 (4)	
H5A	0.5681	0.5531	0.5824	0.021*	
C6	0.7371 (2)	0.49519 (10)	0.62942 (17)	0.0156 (4)	
H6A	0.7064	0.4683	0.5658	0.019*	
C7	1.14826 (18)	0.44366 (9)	0.72276 (16)	0.0120 (3)	
C8	1.1781 (2)	0.49736 (10)	0.66462 (16)	0.0143 (4)	
C9	1.3178 (2)	0.51406 (10)	0.68754 (17)	0.0183 (4)	
H9A	1.3393	0.5497	0.6508	0.022*	
C10	1.4259 (2)	0.47910 (10)	0.76359 (18)	0.0176 (4)	
H10A	1.5178	0.4909	0.7757	0.021*	
C11	1.3966 (2)	0.42688 (10)	0.82107 (17)	0.0162 (4)	
H11A	1.4683	0.4035	0.8725	0.019*	
C12	1.25792 (19)	0.40972 (9)	0.80080 (16)	0.0138 (4)	
H12A	1.2378	0.3749	0.8401	0.017*	
C13	0.9135 (2)	0.39790 (9)	0.54139 (15)	0.0132 (4)	
H13A	0.8412	0.4289	0.5047	0.016*	
H13B	0.9916	0.4068	0.5151	0.016*	
C14	0.72006 (19)	0.33745 (9)	0.34803 (15)	0.0133 (4)	
C15	0.7560 (2)	0.36585 (10)	0.25707 (16)	0.0165 (4)	
C16	0.6482 (2)	0.37698 (12)	0.15341 (17)	0.0233 (5)	
H16A	0.6698	0.3957	0.0930	0.028*	
C17	0.5111 (2)	0.36118 (12)	0.13777 (18)	0.0267 (5)	
H17A	0.4427	0.3688	0.0675	0.032*	
C18	0.4757 (2)	0.33404 (11)	0.22648 (17)	0.0213 (4)	
H18A	0.3837	0.3234	0.2169	0.026*	
C19	0.58066 (19)	0.32297 (11)	0.33074 (16)	0.0167 (4)	
H19A	0.5568	0.3053	0.3909	0.020*	
C20	0.99155 (19)	0.27865 (9)	0.45717 (15)	0.0124 (4)	
C21	0.9691 (2)	0.22669 (10)	0.37912 (16)	0.0146 (4)	
C22	1.0841 (2)	0.20189 (10)	0.35464 (18)	0.0198 (4)	
H22A	1.0705	0.1683	0.3022	0.024*	
C23	1.2183 (2)	0.22580 (12)	0.40620 (19)	0.0244 (5)	
H23A	1.2925	0.2090	0.3871	0.029*	
C24	1.2399 (2)	0.27481 (11)	0.48596 (19)	0.0235 (5)	
H24A	1.3294	0.2904	0.5222	0.028*	
C25	1.1277 (2)	0.30073 (10)	0.51188 (17)	0.0177 (4)	
H25A	1.1432	0.3333	0.5664	0.021*	
C26	1.0458 (2)	0.51396 (11)	0.90788 (18)	0.0226 (4)	
H26A	1.0368	0.5314	0.9770	0.034*	
H26B	1.1193	0.5360	0.8902	0.034*	
H26C	1.0674	0.4689	0.9182	0.034*	
C27	1.0691 (2)	0.53731 (11)	0.57765 (19)	0.0219 (4)	
H27A	1.1129	0.5736	0.5555	0.033*	
H27B	1.0015	0.5522	0.6108	0.033*	
H27C	1.0237	0.5115	0.5114	0.033*	
C28	0.9023 (2)	0.38464 (11)	0.26288 (18)	0.0202 (4)	
H28A	0.8987	0.4074	0.1943	0.030*	
H28B	0.9580	0.3465	0.2698	0.030*	
H28C	0.9428	0.4119	0.3282	0.030*	
C29	0.8274 (2)	0.19827 (10)	0.31925 (17)	0.0175 (4)	
H29A	0.8374	0.1588	0.2823	0.026*	
H29B	0.7729	0.2282	0.2630	0.026*	
H29C	0.7815	0.1898	0.3744	0.026*	
C30	0.79802 (19)	0.36992 (10)	0.84677 (15)	0.0142 (4)	
C31	1.0657 (2)	0.34207 (10)	0.95981 (17)	0.0170 (4)	
C32	1.0840 (2)	0.27162 (9)	0.78005 (16)	0.0141 (4)	
C33	0.63816 (19)	0.32577 (11)	0.62185 (15)	0.0155 (4)	
C34	0.6298 (2)	0.20390 (10)	0.51678 (17)	0.0168 (4)	
C35	0.8977 (2)	0.18882 (10)	0.62918 (16)	0.0158 (4)	
C36	0.6115 (2)	0.26232 (11)	0.81124 (18)	0.0206 (4)	
C37	0.8431 (2)	0.23582 (11)	1.00343 (19)	0.0243 (5)	
C38	0.9560 (2)	0.16876 (11)	0.86278 (18)	0.0198 (4)	
C39	0.4397 (2)	0.12387 (12)	0.65567 (19)	0.0251 (5)	
H39A	0.4861	0.0943	0.6193	0.030*	
H39B	0.4474	0.1671	0.6284	0.030*	
C40	0.2891 (2)	0.10560 (11)	0.62675 (19)	0.0222 (4)	
H40A	0.2432	0.1080	0.5445	0.027*	
H40B	0.2432	0.1354	0.6631	0.027*	
C41	0.6882 (2)	−0.00005 (10)	0.7999 (2)	0.0231 (4)	
H41A	0.5883	−0.0027	0.7622	0.028*	
H41B	0.7099	−0.0160	0.8777	0.028*	
C42	0.7620 (3)	−0.03933 (11)	0.7364 (2)	0.0281 (5)	
H42A	0.7480	−0.0847	0.7480	0.034*	
H42B	0.8611	−0.0306	0.7672	0.034*	
C43	0.5575 (3)	0.07229 (13)	0.9934 (2)	0.0329 (6)	
H43A	0.5991	0.0512	1.0668	0.040*	
H43B	0.5230	0.0391	0.9357	0.040*	
C44	0.4365 (3)	0.11351 (15)	0.9983 (2)	0.0378 (6)	
H44A	0.3706	0.0867	1.0193	0.045*	
H44B	0.3891	0.1313	0.9229	0.045*	

### Atomic displacement parameters (Å^2^)

**Table:**

	*U*^11^	*U*^22^	*U*^33^	*U*^12^	*U*^13^	*U*^23^
Ru1	0.00896 (6)	0.01019 (7)	0.00947 (6)	−0.00107 (5)	0.00188 (5)	0.00001 (5)
Ru2	0.00989 (6)	0.01045 (7)	0.00997 (6)	−0.00169 (5)	0.00238 (5)	−0.00079 (5)
Ru3	0.01346 (7)	0.01283 (7)	0.01105 (7)	−0.00329 (6)	0.00359 (5)	0.00087 (5)
Cl1	0.0268 (3)	0.0272 (3)	0.0268 (3)	−0.0127 (2)	0.0015 (2)	0.0012 (2)
Cl2	0.0782 (6)	0.0479 (4)	0.0265 (3)	0.0071 (4)	0.0162 (3)	0.0035 (3)
Cl3	0.0605 (5)	0.0611 (5)	0.0514 (4)	−0.0233 (4)	0.0316 (4)	−0.0232 (4)
P1	0.0102 (2)	0.0094 (2)	0.0102 (2)	−0.00029 (17)	0.00210 (17)	−0.00042 (16)
P2	0.00951 (19)	0.0108 (2)	0.00951 (19)	0.00008 (17)	0.00197 (16)	−0.00049 (17)
P3	0.0132 (2)	0.0138 (2)	0.0174 (2)	−0.00183 (19)	0.00349 (19)	0.00325 (18)
O1	0.0175 (7)	0.0215 (8)	0.0251 (8)	0.0008 (6)	0.0105 (6)	−0.0009 (6)
O2	0.0252 (8)	0.0373 (9)	0.0151 (7)	−0.0007 (7)	−0.0022 (6)	−0.0044 (7)
O3	0.0179 (7)	0.0183 (8)	0.0275 (8)	0.0005 (6)	0.0084 (6)	−0.0041 (6)
O4	0.0161 (7)	0.0217 (8)	0.0252 (7)	0.0030 (6)	0.0051 (6)	−0.0028 (6)
O5	0.0201 (7)	0.0240 (8)	0.0247 (8)	−0.0077 (6)	0.0025 (6)	−0.0061 (6)
O6	0.0220 (7)	0.0196 (8)	0.0218 (7)	0.0047 (6)	0.0031 (6)	−0.0028 (6)
O7	0.0283 (8)	0.0205 (8)	0.0540 (11)	0.0045 (7)	0.0236 (8)	0.0057 (8)
O8	0.0530 (11)	0.0525 (13)	0.0161 (8)	−0.0167 (10)	0.0097 (8)	−0.0062 (8)
O9	0.0220 (8)	0.0316 (9)	0.0301 (9)	0.0071 (7)	0.0086 (7)	0.0129 (7)
O10	0.0199 (7)	0.0241 (8)	0.0213 (7)	−0.0052 (6)	0.0032 (6)	0.0011 (6)
O11	0.0228 (8)	0.0144 (8)	0.0388 (9)	−0.0035 (6)	0.0146 (7)	−0.0032 (7)
O12	0.0275 (8)	0.0288 (9)	0.0238 (8)	−0.0068 (7)	0.0105 (7)	0.0066 (7)
C1	0.0142 (8)	0.0104 (9)	0.0147 (8)	0.0001 (7)	0.0064 (7)	0.0020 (7)
C2	0.0178 (9)	0.0140 (10)	0.0171 (9)	−0.0012 (7)	0.0064 (8)	0.0010 (7)
C3	0.0216 (10)	0.0136 (10)	0.0211 (10)	−0.0017 (8)	0.0111 (8)	−0.0029 (8)
C4	0.0213 (10)	0.0143 (10)	0.0270 (11)	0.0056 (8)	0.0138 (9)	0.0051 (8)
C5	0.0147 (9)	0.0182 (10)	0.0201 (9)	0.0037 (8)	0.0064 (8)	0.0078 (8)
C6	0.0149 (9)	0.0154 (10)	0.0152 (9)	−0.0008 (7)	0.0033 (7)	0.0011 (7)
C7	0.0104 (8)	0.0135 (9)	0.0127 (8)	−0.0016 (7)	0.0045 (7)	−0.0032 (7)
C8	0.0166 (9)	0.0119 (9)	0.0139 (8)	−0.0007 (7)	0.0044 (7)	−0.0025 (7)
C9	0.0211 (10)	0.0158 (10)	0.0193 (9)	−0.0042 (8)	0.0086 (8)	0.0011 (8)
C10	0.0119 (8)	0.0198 (10)	0.0226 (10)	−0.0034 (7)	0.0077 (8)	−0.0022 (8)
C11	0.0133 (9)	0.0181 (10)	0.0168 (9)	0.0009 (7)	0.0045 (7)	−0.0014 (7)
C12	0.0134 (8)	0.0128 (9)	0.0155 (9)	−0.0011 (7)	0.0052 (7)	−0.0010 (7)
C13	0.0159 (9)	0.0118 (9)	0.0105 (8)	−0.0024 (7)	0.0022 (7)	−0.0004 (7)
C14	0.0146 (8)	0.0126 (10)	0.0102 (8)	0.0014 (7)	0.0008 (7)	−0.0006 (7)
C15	0.0178 (9)	0.0158 (10)	0.0132 (8)	0.0011 (8)	0.0017 (7)	−0.0014 (7)
C16	0.0285 (11)	0.0278 (12)	0.0126 (9)	0.0037 (9)	0.0054 (8)	0.0029 (8)
C17	0.0230 (11)	0.0334 (13)	0.0162 (9)	0.0048 (10)	−0.0037 (8)	0.0025 (9)
C18	0.0159 (9)	0.0231 (12)	0.0205 (9)	0.0023 (8)	0.0001 (8)	−0.0015 (8)
C19	0.0162 (8)	0.0160 (9)	0.0161 (8)	0.0014 (8)	0.0030 (7)	−0.0008 (8)
C20	0.0138 (8)	0.0125 (9)	0.0117 (8)	0.0026 (7)	0.0052 (7)	0.0025 (7)
C21	0.0180 (9)	0.0133 (10)	0.0124 (8)	0.0020 (7)	0.0048 (7)	0.0026 (7)
C22	0.0221 (10)	0.0185 (11)	0.0190 (9)	0.0036 (8)	0.0071 (8)	−0.0022 (8)
C23	0.0161 (9)	0.0305 (12)	0.0287 (11)	0.0081 (9)	0.0102 (8)	0.0009 (9)
C24	0.0121 (9)	0.0309 (13)	0.0269 (11)	0.0008 (8)	0.0058 (8)	−0.0016 (9)
C25	0.0146 (9)	0.0210 (10)	0.0157 (9)	−0.0009 (8)	0.0024 (7)	−0.0014 (8)
C26	0.0204 (10)	0.0223 (11)	0.0196 (10)	0.0031 (9)	−0.0007 (8)	−0.0090 (8)
C27	0.0192 (10)	0.0190 (10)	0.0237 (11)	−0.0020 (8)	0.0020 (8)	0.0089 (8)
C28	0.0225 (10)	0.0224 (11)	0.0166 (9)	−0.0004 (8)	0.0077 (8)	0.0035 (8)
C29	0.0182 (9)	0.0163 (10)	0.0179 (9)	−0.0006 (7)	0.0057 (8)	−0.0043 (7)
C30	0.0117 (8)	0.0161 (10)	0.0126 (8)	−0.0036 (7)	0.0010 (7)	0.0012 (7)
C31	0.0149 (9)	0.0172 (10)	0.0186 (9)	−0.0007 (7)	0.0052 (8)	−0.0009 (7)
C32	0.0155 (9)	0.0126 (10)	0.0125 (8)	−0.0044 (7)	0.0023 (7)	−0.0001 (7)
C33	0.0138 (8)	0.0194 (10)	0.0128 (8)	−0.0050 (8)	0.0036 (7)	−0.0022 (8)
C34	0.0147 (9)	0.0194 (11)	0.0160 (9)	−0.0003 (7)	0.0046 (7)	0.0002 (7)
C35	0.0167 (9)	0.0168 (10)	0.0117 (8)	−0.0041 (8)	0.0016 (7)	−0.0011 (7)
C36	0.0258 (10)	0.0134 (10)	0.0262 (10)	−0.0049 (9)	0.0137 (8)	−0.0003 (8)
C37	0.0266 (11)	0.0261 (12)	0.0212 (11)	−0.0078 (9)	0.0091 (9)	0.0011 (8)
C38	0.0194 (10)	0.0228 (11)	0.0166 (9)	−0.0056 (8)	0.0051 (8)	0.0047 (8)
C39	0.0241 (11)	0.0271 (12)	0.0212 (11)	−0.0020 (9)	0.0035 (9)	0.0032 (9)
C40	0.0178 (10)	0.0223 (11)	0.0231 (11)	−0.0012 (8)	0.0019 (8)	0.0008 (8)
C41	0.0248 (11)	0.0125 (10)	0.0292 (11)	−0.0033 (8)	0.0053 (9)	0.0018 (8)
C42	0.0339 (12)	0.0194 (11)	0.0287 (12)	0.0029 (10)	0.0071 (10)	0.0029 (9)
C43	0.0349 (13)	0.0343 (14)	0.0338 (13)	−0.0105 (11)	0.0170 (11)	0.0080 (11)
C44	0.0358 (14)	0.0510 (18)	0.0310 (13)	−0.0176 (12)	0.0169 (11)	−0.0111 (12)

### Geometric parameters (Å, º)

**Table:**

Ru1—C31	1.890 (2)	C10—C11	1.381 (3)
Ru1—C30	1.931 (2)	C10—H10A	0.9300
Ru1—C32	1.933 (2)	C11—C12	1.395 (3)
Ru1—P1	2.3483 (5)	C11—H11A	0.9300
Ru1—Ru3	2.8415 (2)	C12—H12A	0.9300
Ru1—Ru2	2.8492 (2)	C13—H13A	0.9700
Ru2—C34	1.881 (2)	C13—H13B	0.9700
Ru2—C35	1.931 (2)	C14—C19	1.395 (3)
Ru2—C33	1.933 (2)	C14—C15	1.419 (3)
Ru2—P2	2.3462 (5)	C15—C16	1.404 (3)
Ru2—Ru3	2.8614 (2)	C15—C28	1.517 (3)
Ru3—C37	1.894 (2)	C16—C17	1.382 (3)
Ru3—C38	1.938 (2)	C16—H16A	0.9300
Ru3—C36	1.938 (2)	C17—C18	1.382 (3)
Ru3—P3	2.2543 (5)	C17—H17A	0.9300
Cl1—C40	1.803 (2)	C18—C19	1.395 (3)
Cl2—C42	1.782 (3)	C18—H18A	0.9300
Cl3—C44	1.782 (3)	C19—H19A	0.9300
P1—C7	1.8434 (19)	C20—C25	1.402 (3)
P1—C1	1.844 (2)	C20—C21	1.414 (3)
P1—C13	1.8600 (19)	C21—C22	1.399 (3)
P2—C20	1.8313 (19)	C21—C29	1.507 (3)
P2—C14	1.8518 (19)	C22—C23	1.394 (3)
P2—C13	1.8534 (19)	C22—H22A	0.9300
P3—O11	1.5916 (16)	C23—C24	1.382 (3)
P3—O12	1.6007 (16)	C23—H23A	0.9300
P3—O10	1.6050 (16)	C24—C25	1.392 (3)
O1—C30	1.147 (2)	C24—H24A	0.9300
O2—C31	1.148 (3)	C25—H25A	0.9300
O3—C32	1.152 (2)	C26—H26A	0.9600
O4—C33	1.150 (3)	C26—H26B	0.9600
O5—C34	1.157 (3)	C26—H26C	0.9600
O6—C35	1.143 (3)	C27—H27A	0.9600
O7—C36	1.142 (3)	C27—H27B	0.9600
O8—C37	1.141 (3)	C27—H27C	0.9600
O9—C38	1.151 (3)	C28—H28A	0.9600
O10—C39	1.433 (3)	C28—H28B	0.9600
O11—C41	1.448 (3)	C28—H28C	0.9600
O12—C43	1.439 (3)	C29—H29A	0.9600
C1—C6	1.408 (3)	C29—H29B	0.9600
C1—C2	1.410 (3)	C29—H29C	0.9600
C2—C3	1.401 (3)	C39—C40	1.502 (3)
C2—C26	1.507 (3)	C39—H39A	0.9700
C3—C4	1.383 (3)	C39—H39B	0.9700
C3—H3A	0.9300	C40—H40A	0.9700
C4—C5	1.401 (3)	C40—H40B	0.9700
C4—H4A	0.9300	C41—C42	1.494 (3)
C5—C6	1.384 (3)	C41—H41A	0.9700
C5—H5A	0.9300	C41—H41B	0.9700
C6—H6A	0.9300	C42—H42A	0.9700
C7—C12	1.400 (3)	C42—H42B	0.9700
C7—C8	1.412 (3)	C43—C44	1.516 (4)
C8—C9	1.399 (3)	C43—H43A	0.9700
C8—C27	1.512 (3)	C43—H43B	0.9700
C9—C10	1.392 (3)	C44—H44A	0.9700
C9—H9A	0.9300	C44—H44B	0.9700
			
C31—Ru1—C30	89.14 (8)	H13A—C13—H13B	107.2
C31—Ru1—C32	90.58 (8)	C19—C14—C15	118.81 (17)
C30—Ru1—C32	173.49 (8)	C19—C14—P2	116.59 (14)
C31—Ru1—P1	105.43 (6)	C15—C14—P2	124.59 (14)
C30—Ru1—P1	90.73 (6)	C16—C15—C14	117.61 (19)
C32—Ru1—P1	95.61 (6)	C16—C15—C28	117.38 (19)
C31—Ru1—Ru3	102.45 (6)	C14—C15—C28	125.01 (17)
C30—Ru1—Ru3	80.17 (6)	C17—C16—C15	122.5 (2)
C32—Ru1—Ru3	93.55 (6)	C17—C16—H16A	118.8
P1—Ru1—Ru3	150.492 (13)	C15—C16—H16A	118.8
C31—Ru1—Ru2	160.62 (6)	C18—C17—C16	120.02 (19)
C30—Ru1—Ru2	96.02 (5)	C18—C17—H17A	120.0
C32—Ru1—Ru2	82.20 (5)	C16—C17—H17A	120.0
P1—Ru1—Ru2	93.203 (13)	C17—C18—C19	118.66 (19)
Ru3—Ru1—Ru2	60.372 (6)	C17—C18—H18A	120.7
C34—Ru2—C35	87.53 (8)	C19—C18—H18A	120.7
C34—Ru2—C33	95.85 (8)	C18—C19—C14	122.40 (19)
C35—Ru2—C33	174.03 (8)	C18—C19—H19A	118.8
C34—Ru2—P2	102.19 (6)	C14—C19—H19A	118.8
C35—Ru2—P2	92.71 (6)	C25—C20—C21	119.32 (17)
C33—Ru2—P2	91.38 (6)	C25—C20—P2	119.52 (15)
C34—Ru2—Ru1	165.79 (6)	C21—C20—P2	121.15 (14)
C35—Ru2—Ru1	93.48 (6)	C22—C21—C20	118.00 (18)
C33—Ru2—Ru1	82.04 (5)	C22—C21—C29	118.86 (18)
P2—Ru2—Ru1	91.930 (13)	C20—C21—C29	123.11 (17)
C34—Ru2—Ru3	106.46 (6)	C23—C22—C21	122.2 (2)
C35—Ru2—Ru3	83.32 (6)	C23—C22—H22A	118.9
C33—Ru2—Ru3	91.00 (5)	C21—C22—H22A	118.9
P2—Ru2—Ru3	150.848 (13)	C24—C23—C22	119.33 (19)
Ru1—Ru2—Ru3	59.680 (6)	C24—C23—H23A	120.3
C37—Ru3—C38	91.93 (10)	C22—C23—H23A	120.3
C37—Ru3—C36	93.62 (10)	C23—C24—C25	119.89 (19)
C38—Ru3—C36	173.94 (9)	C23—C24—H24A	120.1
C37—Ru3—P3	98.61 (7)	C25—C24—H24A	120.1
C38—Ru3—P3	90.61 (6)	C24—C25—C20	121.15 (19)
C36—Ru3—P3	86.15 (6)	C24—C25—H25A	119.4
C37—Ru3—Ru1	90.90 (7)	C20—C25—H25A	119.4
C38—Ru3—Ru1	85.24 (6)	C2—C26—H26A	109.5
C36—Ru3—Ru1	97.10 (6)	C2—C26—H26B	109.5
P3—Ru3—Ru1	169.752 (15)	H26A—C26—H26B	109.5
C37—Ru3—Ru2	149.88 (7)	C2—C26—H26C	109.5
C38—Ru3—Ru2	92.86 (6)	H26A—C26—H26C	109.5
C36—Ru3—Ru2	83.55 (6)	H26B—C26—H26C	109.5
P3—Ru3—Ru2	111.042 (14)	C8—C27—H27A	109.5
Ru1—Ru3—Ru2	59.948 (5)	C8—C27—H27B	109.5
C7—P1—C1	105.53 (9)	H27A—C27—H27B	109.5
C7—P1—C13	100.44 (8)	C8—C27—H27C	109.5
C1—P1—C13	103.79 (9)	H27A—C27—H27C	109.5
C7—P1—Ru1	118.08 (6)	H27B—C27—H27C	109.5
C1—P1—Ru1	112.05 (6)	C15—C28—H28A	109.5
C13—P1—Ru1	115.32 (6)	C15—C28—H28B	109.5
C20—P2—C14	104.47 (8)	H28A—C28—H28B	109.5
C20—P2—C13	103.56 (9)	C15—C28—H28C	109.5
C14—P2—C13	99.99 (8)	H28A—C28—H28C	109.5
C20—P2—Ru2	114.25 (6)	H28B—C28—H28C	109.5
C14—P2—Ru2	117.94 (6)	C21—C29—H29A	109.5
C13—P2—Ru2	114.67 (6)	C21—C29—H29B	109.5
O11—P3—O12	106.47 (9)	H29A—C29—H29B	109.5
O11—P3—O10	105.82 (9)	C21—C29—H29C	109.5
O12—P3—O10	95.64 (9)	H29A—C29—H29C	109.5
O11—P3—Ru3	112.45 (6)	H29B—C29—H29C	109.5
O12—P3—Ru3	111.51 (7)	O1—C30—Ru1	172.48 (16)
O10—P3—Ru3	122.83 (6)	O2—C31—Ru1	179.19 (19)
C39—O10—P3	123.43 (14)	O3—C32—Ru1	173.73 (17)
C41—O11—P3	125.37 (14)	O4—C33—Ru2	174.15 (16)
C43—O12—P3	127.12 (16)	O5—C34—Ru2	176.56 (18)
C6—C1—C2	118.54 (18)	O6—C35—Ru2	173.01 (17)
C6—C1—P1	120.05 (15)	O7—C36—Ru3	172.27 (19)
C2—C1—P1	121.25 (15)	O8—C37—Ru3	177.7 (2)
C3—C2—C1	118.49 (18)	O9—C38—Ru3	172.67 (18)
C3—C2—C26	117.62 (18)	O10—C39—C40	107.98 (18)
C1—C2—C26	123.88 (18)	O10—C39—H39A	110.1
C4—C3—C2	122.49 (19)	C40—C39—H39A	110.1
C4—C3—H3A	118.8	O10—C39—H39B	110.1
C2—C3—H3A	118.8	C40—C39—H39B	110.1
C3—C4—C5	119.07 (19)	H39A—C39—H39B	108.4
C3—C4—H4A	120.5	C39—C40—Cl1	109.75 (16)
C5—C4—H4A	120.5	C39—C40—H40A	109.7
C6—C5—C4	119.36 (19)	Cl1—C40—H40A	109.7
C6—C5—H5A	120.3	C39—C40—H40B	109.7
C4—C5—H5A	120.3	Cl1—C40—H40B	109.7
C5—C6—C1	122.00 (19)	H40A—C40—H40B	108.2
C5—C6—H6A	119.0	O11—C41—C42	107.60 (18)
C1—C6—H6A	119.0	O11—C41—H41A	110.2
C12—C7—C8	119.44 (17)	C42—C41—H41A	110.2
C12—C7—P1	116.78 (14)	O11—C41—H41B	110.2
C8—C7—P1	123.74 (14)	C42—C41—H41B	110.2
C9—C8—C7	117.83 (18)	H41A—C41—H41B	108.5
C9—C8—C27	117.79 (18)	C41—C42—Cl2	112.09 (17)
C7—C8—C27	124.38 (18)	C41—C42—H42A	109.2
C10—C9—C8	122.10 (19)	Cl2—C42—H42A	109.2
C10—C9—H9A	119.0	C41—C42—H42B	109.2
C8—C9—H9A	119.0	Cl2—C42—H42B	109.2
C11—C10—C9	120.03 (18)	H42A—C42—H42B	107.9
C11—C10—H10A	120.0	O12—C43—C44	112.3 (2)
C9—C10—H10A	120.0	O12—C43—H43A	109.2
C10—C11—C12	118.98 (18)	C44—C43—H43A	109.2
C10—C11—H11A	120.5	O12—C43—H43B	109.2
C12—C11—H11A	120.5	C44—C43—H43B	109.2
C11—C12—C7	121.60 (18)	H43A—C43—H43B	107.9
C11—C12—H12A	119.2	C43—C44—Cl3	111.89 (19)
C7—C12—H12A	119.2	C43—C44—H44A	109.2
P2—C13—P1	117.51 (10)	Cl3—C44—H44A	109.2
P2—C13—H13A	107.9	C43—C44—H44B	109.2
P1—C13—H13A	107.9	Cl3—C44—H44B	109.2
P2—C13—H13B	107.9	H44A—C44—H44B	107.9
P1—C13—H13B	107.9		
			
C31—Ru1—Ru2—C34	43.1 (3)	C7—P1—C1—C6	131.77 (16)
C30—Ru1—Ru2—C34	−61.6 (3)	C13—P1—C1—C6	26.60 (18)
C32—Ru1—Ru2—C34	112.1 (3)	Ru1—P1—C1—C6	−98.46 (15)
P1—Ru1—Ru2—C34	−152.7 (3)	C7—P1—C1—C2	−52.89 (18)
Ru3—Ru1—Ru2—C34	13.5 (3)	C13—P1—C1—C2	−158.06 (16)
C31—Ru1—Ru2—C35	−50.52 (19)	Ru1—P1—C1—C2	76.88 (16)
C30—Ru1—Ru2—C35	−155.25 (8)	C6—C1—C2—C3	−1.3 (3)
C32—Ru1—Ru2—C35	18.44 (8)	P1—C1—C2—C3	−176.74 (15)
P1—Ru1—Ru2—C35	113.67 (6)	C6—C1—C2—C26	178.31 (19)
Ru3—Ru1—Ru2—C35	−80.18 (6)	P1—C1—C2—C26	2.9 (3)
C31—Ru1—Ru2—C33	125.52 (19)	C1—C2—C3—C4	−0.4 (3)
C30—Ru1—Ru2—C33	20.79 (8)	C26—C2—C3—C4	179.9 (2)
C32—Ru1—Ru2—C33	−165.52 (8)	C2—C3—C4—C5	1.7 (3)
P1—Ru1—Ru2—C33	−70.29 (6)	C3—C4—C5—C6	−1.2 (3)
Ru3—Ru1—Ru2—C33	95.86 (6)	C4—C5—C6—C1	−0.6 (3)
C31—Ru1—Ru2—P2	−143.36 (18)	C2—C1—C6—C5	1.9 (3)
C30—Ru1—Ru2—P2	111.91 (6)	P1—C1—C6—C5	177.35 (15)
C32—Ru1—Ru2—P2	−74.39 (6)	C1—P1—C7—C12	130.80 (15)
P1—Ru1—Ru2—P2	20.834 (17)	C13—P1—C7—C12	−121.58 (15)
Ru3—Ru1—Ru2—P2	−173.017 (13)	Ru1—P1—C7—C12	4.65 (17)
C31—Ru1—Ru2—Ru3	29.66 (18)	C1—P1—C7—C8	−51.49 (18)
C30—Ru1—Ru2—Ru3	−75.07 (6)	C13—P1—C7—C8	56.13 (17)
C32—Ru1—Ru2—Ru3	98.62 (6)	Ru1—P1—C7—C8	−177.64 (13)
P1—Ru1—Ru2—Ru3	−166.149 (13)	C12—C7—C8—C9	0.4 (3)
C31—Ru1—Ru3—C37	17.63 (10)	P1—C7—C8—C9	−177.22 (15)
C30—Ru1—Ru3—C37	−69.28 (9)	C12—C7—C8—C27	179.29 (19)
C32—Ru1—Ru3—C37	109.01 (9)	P1—C7—C8—C27	1.6 (3)
P1—Ru1—Ru3—C37	−143.02 (8)	C7—C8—C9—C10	0.9 (3)
Ru2—Ru1—Ru3—C37	−172.05 (7)	C27—C8—C9—C10	−178.0 (2)
C31—Ru1—Ru3—C38	−74.23 (9)	C8—C9—C10—C11	−1.4 (3)
C30—Ru1—Ru3—C38	−161.14 (8)	C9—C10—C11—C12	0.5 (3)
C32—Ru1—Ru3—C38	17.15 (8)	C10—C11—C12—C7	0.8 (3)
P1—Ru1—Ru3—C38	125.12 (7)	C8—C7—C12—C11	−1.3 (3)
Ru2—Ru1—Ru3—C38	96.09 (6)	P1—C7—C12—C11	176.55 (15)
C31—Ru1—Ru3—C36	111.39 (9)	C20—P2—C13—P1	−103.00 (12)
C30—Ru1—Ru3—C36	24.48 (8)	C14—P2—C13—P1	149.33 (11)
C32—Ru1—Ru3—C36	−157.23 (8)	Ru2—P2—C13—P1	22.14 (13)
P1—Ru1—Ru3—C36	−49.26 (7)	C7—P1—C13—P2	126.57 (11)
Ru2—Ru1—Ru3—C36	−78.29 (6)	C1—P1—C13—P2	−124.43 (11)
C31—Ru1—Ru3—P3	−140.61 (10)	Ru1—P1—C13—P2	−1.50 (13)
C30—Ru1—Ru3—P3	132.49 (10)	C20—P2—C14—C19	135.20 (16)
C32—Ru1—Ru3—P3	−49.23 (10)	C13—P2—C14—C19	−117.87 (16)
P1—Ru1—Ru3—P3	58.74 (9)	Ru2—P2—C14—C19	7.10 (18)
Ru2—Ru1—Ru3—P3	29.71 (8)	C20—P2—C14—C15	−43.81 (19)
C31—Ru1—Ru3—Ru2	−170.32 (6)	C13—P2—C14—C15	63.13 (18)
C30—Ru1—Ru3—Ru2	102.77 (5)	Ru2—P2—C14—C15	−171.90 (14)
C32—Ru1—Ru3—Ru2	−78.94 (5)	C19—C14—C15—C16	−0.9 (3)
P1—Ru1—Ru3—Ru2	29.03 (3)	P2—C14—C15—C16	178.10 (16)
C34—Ru2—Ru3—C37	−160.58 (15)	C19—C14—C15—C28	179.6 (2)
C35—Ru2—Ru3—C37	114.00 (15)	P2—C14—C15—C28	−1.4 (3)
C33—Ru2—Ru3—C37	−64.18 (15)	C14—C15—C16—C17	−0.2 (3)
P2—Ru2—Ru3—C37	30.44 (15)	C28—C15—C16—C17	179.3 (2)
Ru1—Ru2—Ru3—C37	16.00 (14)	C15—C16—C17—C18	0.8 (4)
C34—Ru2—Ru3—C38	100.60 (9)	C16—C17—C18—C19	−0.2 (3)
C35—Ru2—Ru3—C38	15.18 (9)	C17—C18—C19—C14	−0.9 (3)
C33—Ru2—Ru3—C38	−163.00 (8)	C15—C14—C19—C18	1.5 (3)
P2—Ru2—Ru3—C38	−68.38 (7)	P2—C14—C19—C18	−177.58 (17)
Ru1—Ru2—Ru3—C38	−82.82 (7)	C14—P2—C20—C25	128.42 (16)
C34—Ru2—Ru3—C36	−74.50 (9)	C13—P2—C20—C25	24.15 (17)
C35—Ru2—Ru3—C36	−159.92 (8)	Ru2—P2—C20—C25	−101.27 (15)
C33—Ru2—Ru3—C36	21.90 (9)	C14—P2—C20—C21	−52.72 (17)
P2—Ru2—Ru3—C36	116.52 (7)	C13—P2—C20—C21	−157.00 (15)
Ru1—Ru2—Ru3—C36	102.08 (6)	Ru2—P2—C20—C21	77.59 (16)
C34—Ru2—Ru3—P3	8.85 (7)	C25—C20—C21—C22	−3.8 (3)
C35—Ru2—Ru3—P3	−76.57 (6)	P2—C20—C21—C22	177.39 (15)
C33—Ru2—Ru3—P3	105.24 (6)	C25—C20—C21—C29	177.93 (18)
P2—Ru2—Ru3—P3	−160.13 (3)	P2—C20—C21—C29	−0.9 (3)
Ru1—Ru2—Ru3—P3	−174.578 (16)	C20—C21—C22—C23	1.4 (3)
C34—Ru2—Ru3—Ru1	−176.58 (6)	C29—C21—C22—C23	179.8 (2)
C35—Ru2—Ru3—Ru1	98.00 (6)	C21—C22—C23—C24	1.3 (3)
C33—Ru2—Ru3—Ru1	−80.18 (6)	C22—C23—C24—C25	−1.5 (3)
P2—Ru2—Ru3—Ru1	14.44 (3)	C23—C24—C25—C20	−0.9 (3)
C31—Ru1—P1—C7	41.43 (9)	C21—C20—C25—C24	3.6 (3)
C30—Ru1—P1—C7	130.74 (9)	P2—C20—C25—C24	−177.51 (16)
C32—Ru1—P1—C7	−50.72 (9)	C31—Ru1—C30—O1	26.9 (13)
Ru3—Ru1—P1—C7	−158.18 (7)	C32—Ru1—C30—O1	114.4 (14)
Ru2—Ru1—P1—C7	−133.19 (7)	P1—Ru1—C30—O1	−78.5 (13)
C31—Ru1—P1—C1	−81.49 (9)	Ru3—Ru1—C30—O1	129.7 (13)
C30—Ru1—P1—C1	7.82 (9)	Ru2—Ru1—C30—O1	−171.8 (13)
C32—Ru1—P1—C1	−173.65 (9)	C30—Ru1—C31—O2	144 (15)
Ru3—Ru1—P1—C1	78.90 (7)	C32—Ru1—C31—O2	−29 (15)
Ru2—Ru1—P1—C1	103.89 (7)	P1—Ru1—C31—O2	−125 (15)
C31—Ru1—P1—C13	160.09 (9)	Ru3—Ru1—C31—O2	65 (15)
C30—Ru1—P1—C13	−110.61 (9)	Ru2—Ru1—C31—O2	39 (15)
C32—Ru1—P1—C13	67.93 (9)	C31—Ru1—C32—O3	−22.0 (16)
Ru3—Ru1—P1—C13	−39.52 (8)	C30—Ru1—C32—O3	−109.4 (16)
Ru2—Ru1—P1—C13	−14.53 (7)	P1—Ru1—C32—O3	83.6 (16)
C34—Ru2—P2—C20	−88.92 (9)	Ru3—Ru1—C32—O3	−124.5 (16)
C35—Ru2—P2—C20	−0.86 (9)	Ru2—Ru1—C32—O3	176.1 (16)
C33—Ru2—P2—C20	174.80 (9)	C34—Ru2—C33—O4	−46.4 (17)
Ru1—Ru2—P2—C20	92.72 (7)	C35—Ru2—C33—O4	−170.7 (13)
Ru3—Ru2—P2—C20	80.27 (7)	P2—Ru2—C33—O4	56.0 (17)
C34—Ru2—P2—C14	34.39 (9)	Ru1—Ru2—C33—O4	147.8 (17)
C35—Ru2—P2—C14	122.45 (9)	Ru3—Ru2—C33—O4	−153.1 (17)
C33—Ru2—P2—C14	−61.90 (9)	C35—Ru2—C34—O5	−44 (3)
Ru1—Ru2—P2—C14	−143.98 (7)	C33—Ru2—C34—O5	141 (3)
Ru3—Ru2—P2—C14	−156.42 (7)	P2—Ru2—C34—O5	48 (3)
C34—Ru2—P2—C13	151.76 (9)	Ru1—Ru2—C34—O5	−138 (3)
C35—Ru2—P2—C13	−120.18 (9)	Ru3—Ru2—C34—O5	−126 (3)
C33—Ru2—P2—C13	55.47 (9)	C34—Ru2—C35—O6	39.2 (15)
Ru1—Ru2—P2—C13	−26.61 (7)	C33—Ru2—C35—O6	163.9 (12)
Ru3—Ru2—P2—C13	−39.05 (8)	P2—Ru2—C35—O6	−62.9 (15)
C37—Ru3—P3—O11	−117.02 (10)	Ru1—Ru2—C35—O6	−155.0 (15)
C38—Ru3—P3—O11	−24.98 (10)	Ru3—Ru2—C35—O6	146.1 (15)
C36—Ru3—P3—O11	149.90 (10)	C37—Ru3—C36—O7	−73.4 (15)
Ru1—Ru3—P3—O11	40.96 (12)	C38—Ru3—C36—O7	82.8 (18)
Ru2—Ru3—P3—O11	68.33 (7)	P3—Ru3—C36—O7	25.0 (15)
C37—Ru3—P3—O12	2.49 (10)	Ru1—Ru3—C36—O7	−164.8 (15)
C38—Ru3—P3—O12	94.54 (9)	Ru2—Ru3—C36—O7	136.7 (15)
C36—Ru3—P3—O12	−90.59 (9)	C38—Ru3—C37—O8	104 (6)
Ru1—Ru3—P3—O12	160.47 (9)	C36—Ru3—C37—O8	−78 (6)
Ru2—Ru3—P3—O12	−172.16 (7)	P3—Ru3—C37—O8	−165 (6)
C37—Ru3—P3—O10	114.83 (10)	Ru1—Ru3—C37—O8	19 (6)
C38—Ru3—P3—O10	−153.13 (10)	Ru2—Ru3—C37—O8	5 (6)
C36—Ru3—P3—O10	21.74 (10)	C37—Ru3—C38—O9	48.1 (16)
Ru1—Ru3—P3—O10	−87.20 (11)	C36—Ru3—C38—O9	−108.2 (17)
Ru2—Ru3—P3—O10	−59.83 (8)	P3—Ru3—C38—O9	−50.5 (16)
O11—P3—O10—C39	−63.21 (19)	Ru1—Ru3—C38—O9	138.8 (16)
O12—P3—O10—C39	−172.12 (18)	Ru2—Ru3—C38—O9	−161.6 (16)
Ru3—P3—O10—C39	67.74 (18)	P3—O10—C39—C40	170.33 (15)
O12—P3—O11—C41	52.09 (19)	O10—C39—C40—Cl1	−60.7 (2)
O10—P3—O11—C41	−48.89 (19)	P3—O11—C41—C42	169.99 (16)
Ru3—P3—O11—C41	174.49 (15)	O11—C41—C42—Cl2	−70.8 (2)
O11—P3—O12—C43	−82.9 (2)	P3—O12—C43—C44	−87.7 (3)
O10—P3—O12—C43	25.5 (2)	O12—C43—C44—Cl3	−56.7 (3)
Ru3—P3—O12—C43	154.15 (18)		

### Hydrogen-bond geometry (Å, º)

**Table:**

*D*—H···*A*	*D*—H	H···*A*	*D*···*A*	*D*—H···*A*
C11—H11*A*···O1^i^	0.93	2.57	3.204 (3)	126

Symmetry code: (i) *x*+1, *y*, *z*.

## References

[bb1] Bruce, M. I., Liddell, M. J., Hughes, C. A., Patrick, J. M., Skelton, B. W. & White, A. H. (1988*a*). *J. Organomet. Chem.* **347**, 181–205.

[bb2] Bruce, M. I., Liddell, M. J., Shawkataly, O. bin, Hughes, C. A., Skelton, B. W. & White, A. H. (1988*b*). *J. Organomet. Chem.* **347**, 207–235.

[bb3] Bruce, M. I., Shawkataly, O. bin & Williams, M. L. (1985). *J. Organomet. Chem.* **287**, 127–131.

[bb4] Bruker (2009). *APEX2*, *SAINT* and *SADABS* Bruker AXS Inc., Madison, Wisconsin, USA.

[bb5] Cosier, J. & Glazer, A. M. (1986). *J. Appl. Cryst.* **19**, 105–107.

[bb6] Filby, M., Deeming, A. J., Hogarth, G. & Lee, M.-Y. (2006). *Can. J. Chem.* **84**, 319–329.

[bb7] Flack, H. D. (1983). *Acta Cryst.* A**39**, 876–881.

[bb8] Shawkataly, O. bin, Khan, I. A., Hafiz Malik, H. A., Yeap, C. S. & Fun, H.-K. (2011). *Acta Cryst.* E**67**, m197–m198. 10.1107/S1600536811000791 PMC3051575 21522864

[bb9] Shawkataly, O. bin, Khan, I. A., Yeap, C. S. & Fun, H.-K. (2010). *Acta Cryst.* E**66**, m94–m95. 10.1107/S1600536809049927 PMC2980229 21579984

[bb10] Shawkataly, O. bin, Ramalingam, K., Fun, H.-K., Abdul Rahman, A., & Razak, I. A. (2004). *J. Cluster Sci.* **15**, 387–394.

[bb11] Shawkataly, O. bin, Ramalingam, K., Lee, S. T., Parameswary, M., Fun, H.-K. & Sivakumar, K. (1998). *Polyhedron*, **17**, 1211–1216.

[bb12] Sheldrick, G. M. (2008). *Acta Cryst.* A**64**, 112–122. 10.1107/S0108767307043930 18156677

[bb13] Spek, A. L. (2009). *Acta Cryst.* D**65**, 148–155. 10.1107/S090744490804362X PMC2631630 19171970

